# Wie verändern Hybrid-DRGs („diagnosis related groups“) die deutsche Urologie? Eine Umfrage und Potentialanalyse

**DOI:** 10.1007/s00120-024-02515-z

**Published:** 2025-01-20

**Authors:** Philipp Reimold, Christer Groeben, Angelika Borkowetz, Nicole Eisenmenger, Frank König, Marianne Leitsmann, Ulrich Witzsch, Markus Müller, Markus Schöne, Daniela Schultz-Lampel, Margit Fisch, Peter Kollenbach, Andreas Schneider, Jens Westphal, Holger Borchers, Axel Belusa, Maurice Stephan Michel, Björn Volkmer, Johannes Huber

**Affiliations:** 1 Klinik für Urologie, Philipps-Universität Marburg, Baldingerstraße, 35043 Marburg, Deutschland; 2 Urologische Klinik und Poliklinik, Universitätsmedizin Rostock, Rostock, Deutschland; 3 Reimbursement Institute, Hürth, Deutschland; 4 Aturo Praxis Berlin Wilmersdorf, Berlin, Deutschland; 5 Universitätsklinik für Urologie Graz, Graz, Österreich; 6 Urologische Privatpraxis, Med Cube im Limespark, Sulzbach, Deutschland; 7 Urologische Klinik, Klinikum Ludwigshafen, Ludwigshafen, Deutschland; 8 urodocs, Medizinisches Versorgungszentrum für Urologie und Uroonkologie, Speyer, Deutschland; 9 Kontinenzzentrum Südwest, Schwarzwald-Baar Klinikum, Villingen-Schwenningen, Deutschland; 10 Klinik und Poliklinik für Urologie, Universitätsklinikum Hamburg-Eppendorf, Hamburg, Deutschland; 11 Urologie am Weinberg, Urologische Belegabteilung, Elisabeth-Krankenhaus Kassel, Kassel, Deutschland; 12 Facharztpraxis für Urologie, Winsen, Deutschland; 13 Klinik für Urologie, Kinderurologie, Urogynäkologie und Andrologie, Krankenhaus Maria-Hilf, Krefeld, Deutschland; 14 Geschäftsstelle der Deutschen Gesellschaft für Urologie e. V., Berlin, Deutschland; 15 Urologische Gemeinschaftspraxis Chemnitz-Rabenstein, Chemnitz, Deutschland; 16 Klinik für Urologie und Urochirurgie, Universitätsmedizin Mannheim, Mannheim, Deutschland

**Keywords:** Ambulantes Operieren, URS, Sektorengleiche Vergütung, Hydrozele, Ambulantisierung, Outpatient surgery, URS, Equal sector remuneration, Hydrocele, Outpatient treatment

## Abstract

**Hintergrund:**

Mit der Einführung der Hybrid-DRG („diagnosis related groups“) zum 01.01.2024 sollen Anreize geschaffen werden, bisher stationär erbrachte Leistungen in der Urologie, beispielsweise die Ureterorenoskopie (URS), ambulant durchzuführen und sektorengleich zu vergüten. Die Auswirkungen auf die Versorgungsrealität sind derzeit unklar.

**Fragestellung:**

Ziel der Umfrage war es, ein Stimmungsbild zur Einführung der Hybrid-DRG in der Urologie zu erfassen und erste praktische Erfahrungen sowie zukünftige Perspektiven zu beleuchten.

**Material und Methoden:**

In einer deutschlandweiten Online-Umfrage im Zeitraum Mai bis Juli 2024 wurden 32 Fragen u. a. zur Patientenversorgung, Weiterbildung und weiteren Indikationen gestellt. Zudem wurde eine Einschätzung des ambulanten Potenzials für URS bei Harnleiter- und Nierensteinen sowie Hydrozelenresektionen erfragt.

**Ergebnisse:**

Insgesamt beantworteten 364 Urologinnen und Urologen die Umfrage. 54,5 % waren niedergelassen, 45,5 % in Kliniken tätig. 91,1 % waren operativ aktiv. Das Konzept der Hybrid-DRG wurde von 34 % der operativ Tätigen positiv bewertet, jedoch sahen 68 % keine Erleichterung für den Alltag. 51 % äußerten Bedenken hinsichtlich der negativen Auswirkungen auf die Weiterbildung. Der Anteil der 2023 ambulant erbrachten URS lag bei 21 % (Harnleitersteine) und 11 % (Nierensteine) mit langfristigem Steigerungspotenzial von bis zu 33 %. Hydrozelenresektionen wurden bereits zu zwei Dritteln ambulant durchgeführt, und 74 % der Befragten hielten diesen Eingriff für in einer Hybrid-DRG abrechenbar.

**Schlussfolgerung:**

Die Umfrage zeigt ein differenziertes Meinungsbild zu den Hybrid-DRG in der Urologie, jedoch umfasst die Stichprobe nur etwa 6 % der Urologen in Deutschland und Chefärztinnen und Chefärzte sind überrepräsentiert. Eine Wiederholung der Umfrage zu einem späteren Zeitpunkt wäre sinnvoll, um die Entwicklungen im praktischen Einsatz zu evaluieren.

**Graphic abstract:**

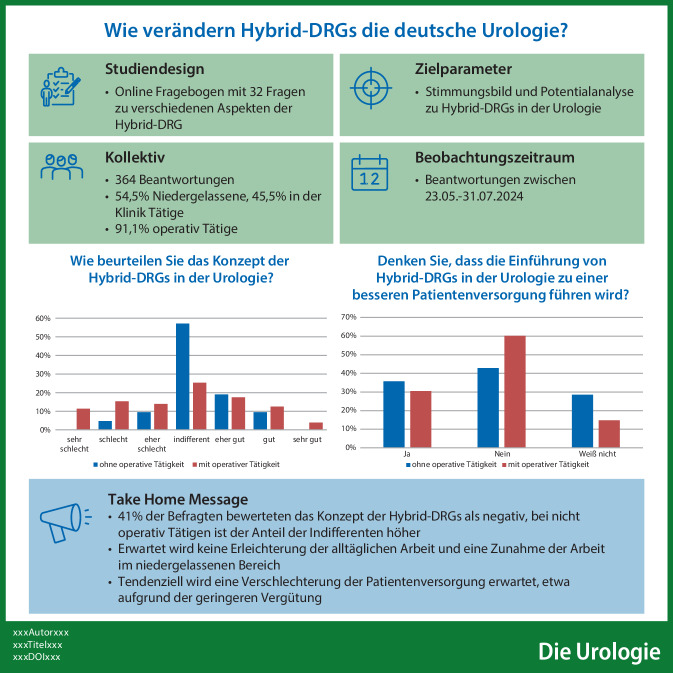

**Zusatzmaterial online:**

Die Online-Version dieses Beitrags (10.1007/s00120-024-02515-z) enthält Zusatzmaterial.

## Abstract (en)

**Background:**

The introduction of hybrid DRGs on 1 January 2024 is intended to create incentives to perform inpatient urology services, e.g., ureterorenoscopy (URS), on an outpatient basis. The effects on the reality of care are currently unclear.

**Objectives:**

The aim of the survey was to gather an opinion on the introduction of hybrid DRGs in urology and to analyze initial practical experiences and future prospects.

**Materials and methods:**

In a Germany-wide online survey conducted between May and July 2024, 32 questions were asked about patient care, further training and other indications, among other things. In addition, an assessment of the outpatient potential for URS for ureteral and kidney stones and hydrocele resections was requested.

**Results:**

A total of 364 urologists responded to the survey: 54.5% were in private practice and 45.5% worked in hospitals. 91.1% were active surgeons. The concept of hybrid DRGs was rated positively by 34% of those working in surgery, but 68% saw no relief in their everyday work. 51% expressed concerns about the negative impact on further training. The proportion of URS performed on an outpatient basis in 2023 was 21% (ureteral stones) and 11% (kidney stones), with a long-term potential increase of up to 33%. Two thirds of hydrocele resections were already performed on an outpatient basis, and 74% of respondents considered these to be billable in a hybrid DRG.

**Conclusion:**

The survey shows a differentiated opinion on hybrid DRGs in urology, but the sample only comprises around 6% of urologists in Germany. It would make sense to repeat the survey at a later date in order to evaluate developments in practical use.

## Kurze Hinführung zum Thema

Die Hybrid-DRG („diagnosis related groups“) sind seit dem 01.01.2024 im Rahmen der sektorengleichen Vergütung des ambulanten und stationären Sektors auch in der Urologie relevant geworden. Die Ureterenoskopie (URS) kann nun sowohl ambulant als auch stationär mit gleichem Erlös erbracht und abgerechnet werden. Im Alltag ergeben sich dadurch viele Fragen: Welche Fälle rechnet man als Hybrid-DRG ab? Wie sind die Verbrauchsmaterialien refinanziert? Wie sind perioperative Leistungen wie Anästhesie, Labor, Pathologie abgebildet? Die folgende Umfrage soll ein Stimmungsbild der deutschen Urologie bezüglich dieses Themas zeichnen.

## Hintergrund

Mit Einführung der Hybrid-DRG zum 01.01.2024 sollen durch eine sektorengleiche Vergütung Anreize geschaffen werden, bisher stationär erbrachte Leistungen ambulant zu erbringen. Ziel ist die Förderung der Ambulantisierung bislang stationär durchgeführter Operationen und somit die Entlastung des stationären Bereichs insbesondere auch durch Entlastung des Pflegepersonals [1]. In der Urologie ist die Ureterorenoskopie aktuell der Leistungsbereich, der nach Hybrid-DRG abgerechnet werden kann. Die Auswirkungen auf die Versorgungsrealität unserer Patientinnen und Patienten sind zum jetzigen Zeitpunkt völlig unklar.

## Fragestellung

Das Ziel unserer Umfrage war es daher, ein Stimmungsbild über die Einführung der Hybrid-DRG in der Urologie einzuholen und erste praktische Erfahrungen zu erheben. Auch die zukünftigen Perspektiven der Hybrid-DRG für unser Fachgebiet sollten beleuchtet werden.

## Studiendesign und Untersuchungsmethoden

In einem gemeinsamen Projekt der Deutschen Gesellschaft für Urologie e. V. (DGU) und des Berufsverbands der Deutschen Urologie e. V. (BvDU), ausgehend vom AK Versorgungsforschung, Qualität und Ökonomie und der AG Sektorenübergreifende Fachärztliche Urologische Versorgung erstellten wir einen Fragebogen zur Einführung der Hybrid-DRG in der Urologie mittels SurveyMonkey® (SurveyMonkey Inc., San Mateo, CA, USA, www.surveymonkey.de, Fragebogen siehe Zusatzmaterial in der Online-Version).

Die Versendung der Online-Umfrage erfolgte über die Mailverteiler der DGU und der BvDU. Die Beantwortung war vom 23.05.–31.07.2024 möglich.

Insgesamt enthielt der Fragebogen 32 Fragen mit unterschiedlicher dynamischer Struktur für operativ und nicht operativ Tätige. Methodisch wurden Bewertungen anhand einer 7‑stufigen Likert-Skala erhoben. Des Weiteren waren Freitextantworten möglich, die in der Auswertung geclustert und nach Kategorien sortiert wurden. Der nicht validierte Fragebogen wurde in mehreren Schritten mit den Mitgliedern des AK Versorgungsforschung, Qualität und Ökonomie und der AG Sektorenübergreifende fachärztliche urologische Versorgung entwickelt und nach einer ersten Testphase optimiert.

Die statistische Analyse erfolgte mit SPSS (IBM Corp. IBM SPSS Statistics for Macintosh, Version 29.0.2.0 Armonk, NY, IBM Corp). Da keine direkten Gruppenvergleiche durchgeführt wurden, erfolgte dies deskriptiv, es wurde keine Signifikanzprüfung berechnet.

## Ergebnisse

### Soziodemographie und Beschäftigungsstruktur

Insgesamt 364 Urolog*innen beantworteten unsere Umfrage, davon 54,5 % Niedergelassene und 45,5 % primär an einer Klinik Tätige. Das mittlere Alter lag bei 51 (43–58) Jahren, 79,5 % davon männlich, 19,9 % weiblich. Die regionale Verteilung der Antwortenden zeigte eine Konzentration in Ballungsräumen mit einem Ost-West-Gefälle zugunsten der alten Bundesländer.

An einer Klinik Beschäftigte arbeiteten v. a. in Häusern der Schwerpunktversorgung (33,3 %) und Regelversorgung (26,2 %), hauptsächlich in kommunaler (32,3 %) oder frei-gemeinnütziger (30,4 %) Trägerschaft.

**Abb. 1:**
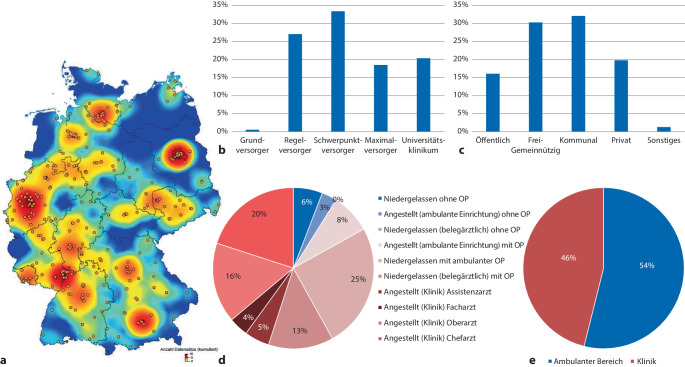
Zusammensetzung der Kohorte: **a** Räumliche Verteilung der Antworten. Ein *Kreis* entspricht einer Beantwortung (Abbildung erstellt mit EasyMap V11.1). Verteilung der in einer Klinik Angestellten nach Versorgungsniveau (**b**) und Trägerschaft (**c**). Verteilung der Antworten nach Art der ärztlichen Tätigkeit (**d**). In *blauen* Farben nicht operativ Tätige, in *roten* Farben operativ Tätige. Verteilung der Antworten nach ambulantem und stationärem Bereich (**e**). Zur besseren Übersichtlichkeit wird nur die männliche Form dargestellt, es sind ausdrücklich alle Kolleg*innen gemeint

Von allen Teilnehmenden waren 91,1 % auch selbst operativ tätig. Die größte Gruppe stellten mit 24,9 % Niedergelassene mit operativer Tätigkeit dar (z. B. ambulantes OP-Zentrum), gefolgt von Chefärzt*innen an einer Klinik (20,1 %). Die regionale Verteilung und genaue Verteilung nach ärztlicher Tätigkeit, Krankenhausform und Trägerschaft sind in Abb. 1 zusammengefasst.

## Beurteilung der Hybrid-DRG in der Urologie

Auf die allgemeine Frage „Wie beurteilen Sie das Konzept der Hybrid-DRG in der Urologie?“ bewerteten operativ tätige Teilnehmer das Konzept der Hybrid-DRG zu 34 % positiv, 25 % waren indifferent und 41 % negativ. In der Gruppe der nicht operativ Tätigen war der Anteil der Indifferenten wesentlich höher (57 %).

Weiter sahen in der Gruppe mit operativer Tätigkeit 68 % durch die Einführung der Hybrid-DRG keine Erleichterung für ihre alltägliche Arbeit – bei denen ohne operative Tätigkeit lag dieser Anteil bei 35 %, mehr als die Hälfte jedoch (55 %) votierten indifferent.

Die Frage der Gerechtigkeit des neuen Abrechnungskonzepts wurde mit der Frage „Ich finde es gerecht, dass derselbe Eingriff sowohl ambulant als auch stationär über die Hybrid-DRG abgerechnet werden kann“ evaluiert. Hier zeigten sich die Unterschiede zwischen operativ und nicht operativ Tätigen weniger markant: 34 % (nicht operativ: 23 %) stimmten dieser Aussage nicht zu, indifferent zeigten sich 13,3 % (nicht operativ: 23,8 %), Zustimmung fand sich bei 62,4 % (52,4 %).

**Abb. 2:**
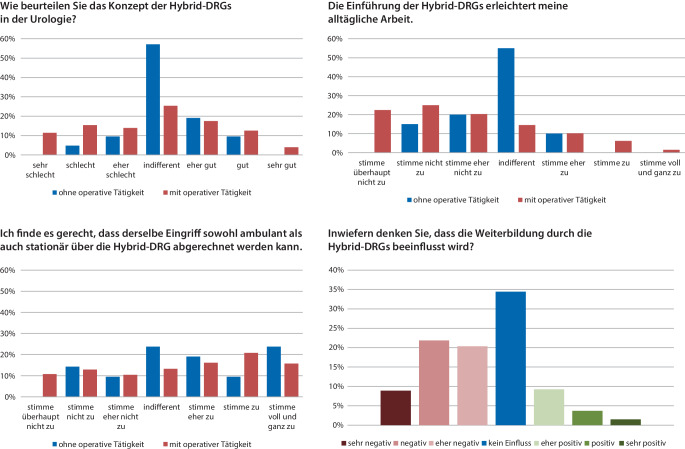
Beurteilung der Hybrid-DRG in der Urologie: prozentuale Anteile der Antworten auf die gestellten Fragen getrennt nach operativer und nicht operativer Tätigkeit. Die Frage nach Einfluss auf die Weiterbildung wurde der gesamten Kohorte ohne Unterscheidung gestellt

Der Einfluss der Einführung von Hybrid-DRG auf die Weiterbildung des urologischen Nachwuchses wurde von 51 % als negativ bewertet, 34 % waren indifferent. Die detaillierte Darstellung der Beantwortungen der oben genannten Fragen sind in Abb. 2 zusammengefasst.

## Einfluss auf nicht (mehr) operativ tätige Niedergelassene

**Abb. 3:**
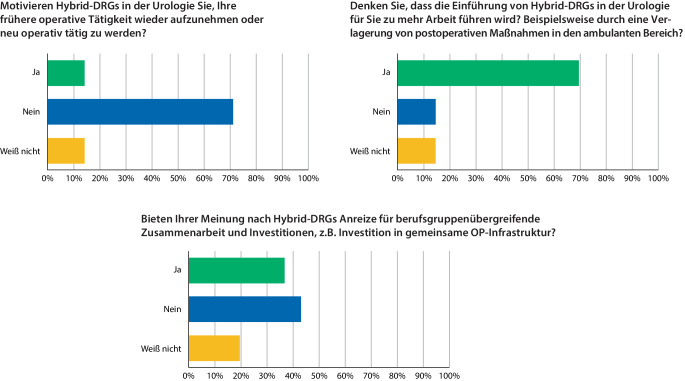
Spezifische Fragen zur operativen Tätigkeit, Arbeitsbelastung und Investitionen: Neben der Motivation, (wieder) operativ tätig zu werden und der Einschätzung zu zusätzlicher Arbeitsbelastung wurde auch abgefragt, ob Hybrid-DRG Anreize für Investitionen sein könnten

In der Kohorte der nicht (mehr) operativ tätigen Urologinnen und Urologen wurde die Frage, ob die Einführung der Hybrid-DRG sie motivieren würde, neu operativ tätig zu werden oder eine frühere operative Tätigkeit wieder aufzunehmen vom Großteil (71 %) mit „Nein“ beantwortet. Etwa derselbe Anteil (70 %) fürchtete die Entstehung von Mehrarbeit, beispielsweise durch Verlagerung von postoperativen Maßnahmen in den ambulanten Bereich. Für die operativ Tätigen ergab sich bei 43,3 % kein Anreiz für berufsgruppenübergreifende Zusammenarbeit und Investitionen z. B. in gemeinsame OP-Infrastruktur, 37 % sahen diese Anreize gegeben (Abb. 3).

## Auswirkungen auf die Patientenversorgung

**Abb. 4:**
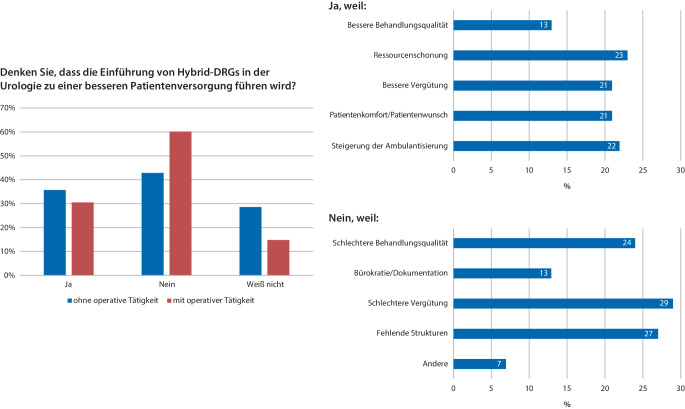
Einschätzung zum Einfluss auf die Patientenversorgung: Die Freitextantworten wurden ausgewertet, gruppiert und in die oben genannten Hauptkategorien sortiert

Ob die Einführung von Hybrid-DRG in der Urologie zu einer besseren Patientenversorgung führen wird, beantworteten ca. ein Drittel der Befragten mit „Ja“ (nicht operativ tätig: 35,7 %, operativ tätig 30,5 %), die Mehrheit (nicht operativ tätig: 42,9 %, operativ tätig 60,2 %) jedoch mit „Nein“. Die Freitextantworten auf diese Frage (*n* = 249) wurden kategorisiert und zusammengefasst. In der Gruppe, die eine Verbesserung der Patientenversorgung erwartet, waren 23 % der Auffassung, dass eine Schonung von Ressourcen wie z. B. Krankenhausbetten sich positiv auswirken wird. Andere Gründe waren die Entsprechung der Patientenwünsche (etwa kurzfristigere Termine, Nachbehandlung durch Operateur, Vermeidung von Krankenhausübernachtungen durch ambulante Operationen) und eine bessere Vergütung (je 21 %) sowie eine als positiv bewertete Zunahme der Ambulantisierung (23 %) und die Steigerung der Behandlungsqualität (13 %). Im Gegensatz dazu bewerteten in der Gruppe derer, die keine Verbesserung der Patientenversorgung erwarteten die Behandlungsqualität als schlechter (24 %), die Vergütung als geringer (29 %). Weitere negativ konnotierte Punkte waren der Dokumentationsaufwand (13 %) und die Einschätzung, dass für die mit der Einführung der Hybrid-DRG in der Urologie einhergehende zunehmende Ambulantisierung die Strukturen (personell, räumlich, technisch) nicht vorhanden seien (27 %). Die Ergebnisse zur Einschätzung des Einflusses der Hybrid-DRG auf die Patientenversorgung sind in Abb. 4 zusammengefasst.

## Mögliche Ausweitung der Hybrid-DRG in der Urologie

**Abb. 5:**
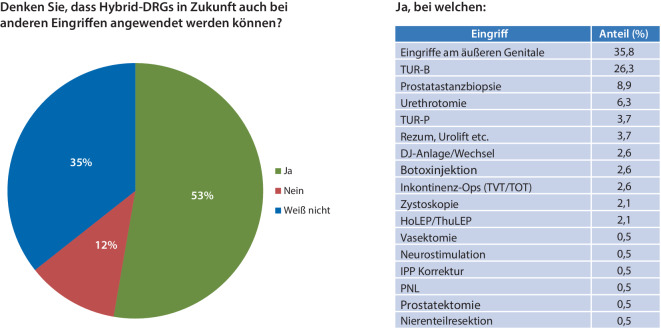
Mögliche Zukunft der Hybrid-DRG in der Urologie: Die Freitextantworten wurden ausgewertet, gruppiert und in die oben genannten Hauptkategorien sortiert (*TVT* „tension-free vaginal tape“, *TOT* „trans-obturator-tape“, *HoLEP* Holmium-Laserenukleation der Prostata, *ThuLEP* Thulium-Laserenukleation der Prostata, *IPP* Induration penis plastica, *PNL* perkutane Nephrolitholapaxie)

Mehr als die Hälfte der Befragten (53 %) gaben an, dass Hybrid-DRG auch bei anderen urologischen Eingriffen angewendet werden könnten. Vor allem Eingriffe am äußeren Genitale (35,8 %) und die TUR-Blase (26,3 %) wurden als denkbare sektorengleich zu vergütende Eingriffe genannt (Abb. 5).

## Potentialanalyse der ambulanten Steigerung

**Abb. 6:**
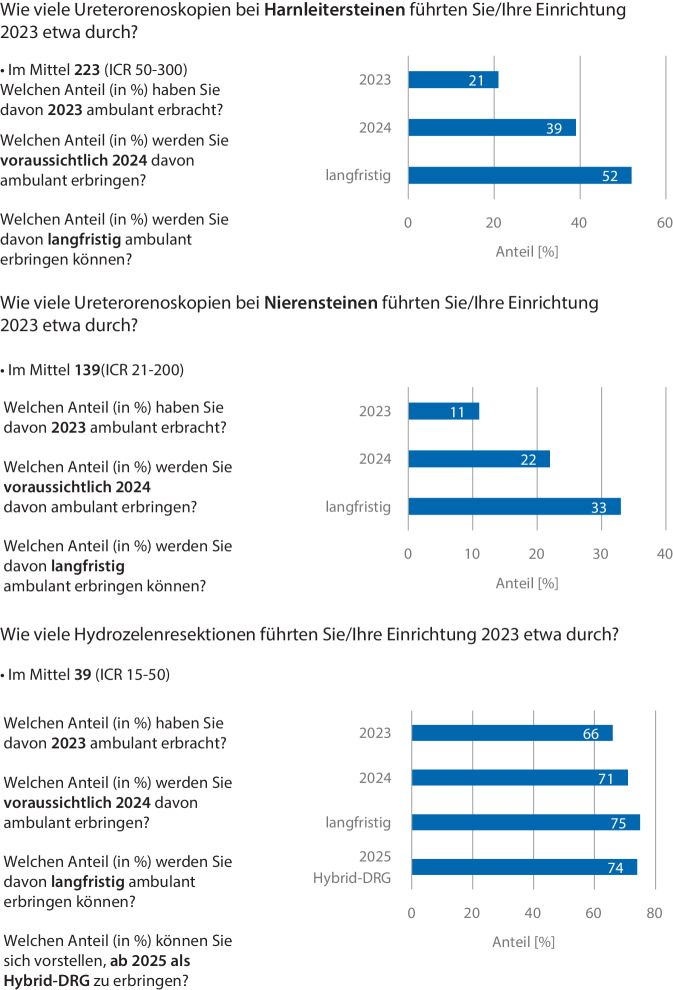
Potentialanalyse zur URS und Hydrozelenresektion: Neben der Anzahl an durchgeführten Eingriffen wurde der Anteil ambulant erbrachter Eingriffe erfasst; des Weiteren die voraussichtliche Entwicklung im Jahr 2024 und langfristig. Für die Hydrozelenresektion wurde außerdem die Bereitschaft, diesen Eingriff als Hybrid-DRG abzubilden, abgefragt (*IQR* „interquartile range“)

Im Jahr 2023 wurden von den Teilnehmenden 21 % der URS bei Harnleitersteinen ambulant erbracht – für die URS bei Nierensteinen waren es 11 %, für Hydrozelenresektionen 66 %. Der voraussichtliche Anteil an ambulant erbrachten Eingriffen im Jahr 2024 betrug für die URS bei Harnleitersteinen 39 % (+18 %), die URS bei Nierensteinen 22 % (+11 %) und die Hydrozelenresektion 71 % (+5 %). Das langfristige ambulante Potenzial wurde für die URS bei Harnleitersteinen mit 52 % (+31 %), die URS bei Nierensteinen mit 33 % (+22 %) und die Hydrozelenresektion mit 75 % (+9 %) angegeben. Des Weiteren betrug das geschätzte ambulante Potenzial der Hydrozelenresektion im Rahmen einer Hybrid-DRG ab 2025 74 % (+8 %). Die detaillierte Potentialanalyse zeigt Abb. 6.

## Diskussion

Unsere Umfrage ist die deutschlandweit erste in der Urologie zur Beurteilung der Hybrid-DRG in der betroffenen Community und liefert wichtige Erkenntnisse über das Stimmungsbild innerhalb der deutschen Urologie, aber auch mögliche zukünftige Implikationen.

Das befragte Kollektiv entspricht ca. 6 % der deutschen Urologinnen und Urologen mit einem Großteil (91 %) an operativ Tätigen. Die Verteilung der Antworten zwischen ambulantem (55 %) und stationärem (45 %) Sektor entspricht in etwa der Literatur, nach welcher 2020 52 % der Kolleg*innen im ambulanten Sektor beschäftigt waren [2]. Mit 20 % Anteil an Antworten ist die Gruppe der Chefärzt*innen deutlich überrepräsentiert. Auch wenn das Kollektiv keine repräsentative Stichprobe der gesamten deutschen urologischen Versorgungsstruktur darstellt, stammen die Antworten doch schwerpunktmäßig aus der Gruppe der Betroffenen. Hierzu gehört in den Kliniken sicher auch die überproportionale Beteiligung der strategischen Entscheidungsträger. Weiterhin ist zu bemerken, dass sich die direkten ökonomischen Effekte der Einführung der Hybrid-DRG v. a. auf die Chefärzt*innen sowie belegärztlich Tätige und Niedergelassene, die in OP-Zentren operieren, auswirken werden. Die anderen befragten Gruppen werden eher von indirekten Auswirkungen, wie z. B. Veränderung der Weiterbildung, der Personalsituation oder der Verlagerung der postoperativen Maßnahmen, betroffen sein.

Eine Limitation der Umfrage besteht in der Tatsache, dass der versendete Fragebogen nicht validiert ist. Dies birgt die Gefahr, dass die Struktur und Formulierung der Fragen einen Einfluss auf die Beantwortung haben. Daher wurde der Fragebogen durch die Mitglieder der AK Versorgungsforschung, Qualität und Ökonomie und der AG Sektorenübergreifende fachärztliche urologische Versorgung in mehreren Schritten entwickelt, und nach einer ersten Testphase optimiert. Um erste Erfahrungen sowie das Stimmungsbild im Kontext der Hybrid-DRG in der Urologie zu erfassen, erscheint uns dies ausreichend.

Auch liegt der Umfragezeitpunkt in einer frühen Phase der Einführung dieses Modells, sodass die praktischen Erfahrungen noch limitiert sein könnten. Die Eindrücke entstammen den Abrechnungen des ersten Quartals. Die Wiederholung der Umfrage zu einem späteren Zeitpunkt – ergänzt um bis dahin neue Aspekte – wäre daher sicher lohnend.

Die Beurteilung der Hybrid-DRG auf der siebenstelligen Likert-Skala ergab eine negative Bewertung bei 41 % der operativ Tätigen. Diese Frage kann einen Trend im Sinne eines Stimmungsbildes vermitteln, allerdings keine Analyse der Gründe. Einzelne Aspekte der Auswirkungen der Hybrid-DRG wurde gezielt erfragt, u. a. die Auswirkung auf die Weiterbildung sowie die Erleichterung der alltäglichen Arbeit und die Frage der Gerechtigkeit einer sektorengleichen Vergütung. Die Bewertung der Hybrid-DRG entspricht dem Trend, der in Umfragen anderer Fachgesellschaften erhoben wurde. Der Berufsverband der Anästhesistinnen und Anästhesisten e. V. (BDA) veröffentlichte eine Umfrage, in der 75 % der Befragten das Konzept als „schlecht“ oder „sehr schlecht“ bewerteten [3]. Hauptsächlich wurden von den Kolleg*innen Einbußen im Bereich der Honorare befürchtet.

In einer Stellungnahme des Berufsverbands der Deutschen Chirurgie e. V. (BDC) wurden neben der Honorierung insbesondere die Aufteilung der Leistungen zwischen operierender Abteilung und Anästhesie (z. B. Laborleistungen), die an der Realität scheiternde Sachkostenkalkulation und die fehlende Existenz der Abrechnungsstrukturen für eine Hybrid-DRG im vertragsärztlichen Bereich bemängelt [4].

In der gezielten Befragung der Kolleg*innen im nicht operativ ambulanten Bereich ergab unsere Umfrage, dass der Großteil (71 %) durch die Hybrid-DRG nicht motiviert wird (wieder) operativ tätig zu werden. Außerdem wird eine deutliche Mehrarbeit, z. B. durch Verlagerung von Maßnahmen (z. B. Katheterzug, Verlaufssonographien, Wundkontrollen) in den ambulanten Bereich von 70 % der Befragten befürchtet. Eine stärkere Ambulantisierung durch eine zunehmende Zahl ambulant operativ tätiger Urolog*innen ist auf dem Boden dieser Ergebnisse daher nicht zu erwarten. Dies widerspricht der Vorstellung, dass eine Zunahme der Ambulantisierung nur durch mehr „Hybridärzte, die vormittags ambulant operieren oder andere ambulantisierte Leistungen erbringen und nachmittags in der Praxis arbeiten“ zu erreichen sei [5].

Eine der Kernfragen, an der sich das Konzept der Hybrid-DRG in der Urologie messen lassen muss, ist die nach der Verbesserung der Patientenversorgung. Das befragte Kollektiv schätzte diese Verbesserung zum Großteil als unrealistisch ein und lieferte dafür folgende Gründe: Neben der erwarteten schlechteren Behandlungsqualität und dem Dokumentationsaufwand sind v. a. die schlechte Honorierung und die fehlenden Strukturen im ambulanten Bereich Kritikpunkte.

Grundsätzlich müssen die indikationsgerecht erbrachten Leistungen auch gerecht vergütet werden, d. h. sowohl im ambulanten als auch im stationären Bereich muss eine kostendeckende Erbringung möglich sein. Als Anreiz kann man im ambulanten Setting dann mit etwas höheren Gewinnen rechnen. Insbesondere das sehr kostenintensive Einmalmaterial zur Steintherapie ist in der aktuellen Bemessung nicht ausreichend berücksichtigt. Diese Unterdeckung könnte kurzfristig dazu führen, dass niemand mehr diese häufigen Steintherapien erbringen möchte, was die Versorgung massiv verschlechtern könnte. Zur Sachkostenabrechnung im Zusammenhang mit der Hybrid-DRG äußert sich Dr. Weinhart vom Spitzenverband Fachärzte Deutschlands e. V. (SpiFA) wie folgt: „Um die gewünschten Effekte der Ambulantisierung wirken zu lassen, wird [daher] dringend vorgeschlagen, die Sachkosten aus der Fallpauschale herauszunehmen“ [6].

Bezüglich der fehlenden Strukturen, die eine sektorengleiche Vergütung realisierbar erscheinen lassen, ist anzumerken, dass im Rahmen der Krankhausstrukturreform vorgesehen ist, dass Mittel für den Aufbau hybrider Versorgungsstrukturen genutzt werden dürfen – diese Finanzierungsmöglichkeit im ambulanten Bereich jedoch fehlt [6]. Die Abrechnungslogik und -strukturen (z. B. Grouping-Software, Kodierungsfachkräfte), die es in den Kliniken seit Jahren gibt, fehlen im ambulanten Bereich größtenteils und können nicht ohne Weiteres aufgebaut werden [7]. Ob die Krankenkassen bis zum Ende der Übergangsregelung zum 01.01.2025 dazu in der Lage sein werden, die eingehenden Rechnungen nach den vorgegebenen 21 Tagen zu begleichen, ist fraglich, da die Infrastruktur des elektronischen Abrechnungsverfahrens erst noch etabliert werden muss.

Laut den Ergebnissen unserer Umfrage besteht bei den bereits über eine Hybrid-DRG abbildbaren Eingriffe der URS bei Harnleiter- oder Nierensteinen ein signifikantes ambulantes Steigerungspotential von bis zu 31 %. Inwiefern der Ambulantisierungsgrad in der Urologie durch die Einführung der Hybrid-DRG zunehmen wird, ist zum jetzigen Zeitpunkt nicht genauer abzuschätzen.

Die Einschätzung, dass u. a. Eingriffe am äußeren Genitale dazu geeignet wären, als Hybrid-DRG abgerechnet zu werden, deckt sich mit den in der Zwischenzeit bekanntgewordenen Erweiterungen des Hybrid-DRG-Katalogs zum 01.01.2025, in dem u. a. die Hydrozelenresektion, Orchidopexie, Varikozelenresektion und Epididymektomie aufgenommen wurden. Erfreulicherweise wurden hier seitens der Verhandlungspartner die Empfehlungen der AG Sektorenübergreifende fachärztliche Versorgung der DGU berücksichtigt. Für etwaige weitere Verhandlungen erscheint in diesem Kontext interessant, dass auch die TURB von 26 % derer als sektorengleich vergütbar benannt wurde, die eine Erweiterung der Hybrid-DRG auf andere Eingriffe für möglich hielten. Da bei diesem Eingriff der Komplexitätsgrad stark schwanken kann, ist hierbei jedoch sicher mit Einschränkungen zu rechnen. Denkbar wären TURB „einfacherer“ Tumoren oder Nachresektionen ohne photodynamische Diagnostik.

Zusammenfassend liefert die präsentierte Umfrage ein wichtiges Stimmungsbild zur Einführung der Hybrid-DRG in der Urologie. Die überwiegend negative Bewertung des Abrechnungsmodus ist sicher auch der Art der Einführung geschuldet, nämlich per Rechtsverordnung durch das Bundesministerium für Gesundheit und ohne längere Übergangsfristen. Nach einigen Klarstellungen und Nachbesserungen erscheint das Konzept zumindest in einigen Indikationen als praktikabel. Nichtsdestotrotz muss die Frage der Honorierung, insbesondere in Bezug auf die Sachkosten, diskutiert und korrigiert werden. Sollten keine ausreichenden Anreize geschaffen werden, Steinsanierungen ambulant zu erbringen, könnte dies gravierende Auswirkungen auf die Versorgungsituation in Deutschland haben. Eine Wiederholung unserer Umfrage nach mehreren Abrechnungsquartalen und inklusive der neu hinzugekommenen Indikationen erscheint somit sinnvoll.

## Fazit für die Praxis

- Aktuell können die Ureterorenoskopie (URS) bei Harnleiter- und Nierensteinen als Hybrid-DRG („diagnosis related groups“) abgerechnet werden.
- Die kostendeckende Erbringung dieser Eingriffe scheitert an der mangelnden Berücksichtigung der Sachkosten.
- Die Einschätzung der deutschen urologischen Community ist daher eher negativ.
- Insbesondere befürchtet wird eine Verschiebung von perioperativen Aufgaben in den niedergelassenen Bereich.

## Supplementary Information

Supplementary Fig. 1

## Data Availability

Die Daten sind auf begründete Anfrage bei dem korrespondierenden Autor erhältlich.
