# Supplementary material for: How will hybrid-DRGs (diagnosis related groups) change German urology? A survey and potential analysis
Source: Urologie. 2025 Jan 20;64(5):453–61. [Article in German] doi: 10.1007/s00120-024-02515-z (PMC12041156; doi:10.1007/s00120-024-02515-z)
Supplement: Supplementary file 1 — Supplementary Fig. 1 [file 120_2024_2515_MOESM1_ESM.pdf]

Zusatzmaterial: https://doi.org/10.1007/s00120-024-02515-z

Hybrid-DRGs in der Urologie

Hybrid-DRGs in der Urologie

Sehr geehrte Frau Kollegin, sehr geehrter Herr Kollege,

die Einführung von Hybrid-DRGs in der Urologie verändert die Art der Abrechnung für uns alle. Perspektivisch werden außer der Ureterorenoskopie sicher auch noch andere urologische Eingriffe über eine Hybrid-DRG abgerechnet werden.

Ziel der folgenden Umfrage ist es, ein Stimmungsbild zu erhalten und Ihre persönliche Versorgungsrealität zu erfassen. Außerdem sind wir für Ihre persönliche Bewertung der aktuellen und zukünftigen Situation mit Hybrid-DRGs in der Urologie dankbar.

Der Fragebogen wird nicht mehr als 10 Minuten in Anspruch nehmen. Wir bedanken uns ganz herzlich für Ihre Teilnahme!

Die Ergebnisse der Umfrage werden auf dem diesjährigen DGU-Kongress in Leipzig präsentiert.

Vielen Dank für Ihr Engagement!

Mit kollegialen Grüßen,

Prof. Dr. med. Maurice Stephan Michel
Generalsekretär der Deutschen Gesellschaft für Urologie e.V.
Dr. med. Axel Belusa
Präsident des Berufsverbandes der Deutschen Urologie e.V.
Prof. Dr. med. Dr. phil. Johannes Huber
AK Versorgungsforschung, Qualität und Ökonomie der DGU
Prof. Dr. med. Markus Müller und Dr. Markus Schöne
AG Sektorenübergreifende fachärztliche urologische Versorgung

Zusatzmaterial: https://doi.org/10.1007/s00120-024-02515-z

Hybrid-DRGs in der Urologie

1. Wie alt sind Sie (in Jahren)?

2. Welches Geschlecht haben Sie?

- Männlich
- Weiblich
- Divers

3. Bitte nennen Sie uns die PLZ Ihrer beruflichen Tätigkeit:

Zusatzmaterial: https://doi.org/10.1007/s00120-024-02515-z

Hybrid-DRGs in der Urologie

4. Welche Beschreibung der ärztlichen Tätigkeit kommt Ihrer am nächsten?

- ○ Niedergelassen ohne ambulante OP-Tätigkeit
- ○ Angestellt in einer ambulanten Einrichtung (Praxis, MVZ, o.ä.) **mit** operativer Tätigkeit
- ○ Angestellt in einer ambulanten Einrichtung (Praxis, MVZ, o.ä.) **ohne** operative Tätigkeit
- ○ Niedergelassen mit ambulanter OP-Tätigkeit
- ○ Niedergelassen mit belegärztlicher Tätigkeit und **mit** operativer Tätigkeit
- ○ Niedergelassen mit belegärztlicher Tätigkeit **ohne** operative Tätigkeit
- ○ Angestellt in einer Klinik als Assistenzärztin/Assistenzarzt
- ○ Angestellt in einer Klinik als Fachärztin/Facharzt
- ○ Angestellt in einer Klinik als Oberärztin/Oberarzt
- ○ Angestellt in einer Klinik als Chefärztin/Chefarzt

Zusatzmaterial: https://doi.org/10.1007/s00120-024-02515-z

Hybrid-DRGs in der Urologie

5. An welcher Art von Klinikum arbeiten Sie?

- ◯ Grundversorger (Abteilung f. Inneren Medizin und/oder Chirurgie)
- ◯ Regelversorger (Innere Medizin und Chirurgie + weitere Fachabteilung)
- ◯ Schwerpunktversorger (obige + Kinderheilkunde und/oder Neurologie)
- ◯ Maximalversorger (alle Fachabteilungen)
- ◯ Universitätsklinikum (Maximalversorger + Lehre + Forschung)

Zusatzmaterial: https://doi.org/10.1007/s00120-024-02515-z

Hybrid-DRGs in der Urologie

6. Zu welcher Gruppe zählt der Träger Ihrer Einrichtung?

- ◯ Öffentlicher Träger (z.B. Universitätskliniken)
- ◯ Frei-Gemeinnütziger Träger (z.B. DRK, kirchliche Häuser)
- ◯ Kommunaler Träger (Stadt, Landkreis)
- ◯ Privater Träger (z.B. Sana, Helios, Asklepios…)
- ◯ Privatklinik
- ◯ Sonstiges

Zusatzmaterial: https://doi.org/10.1007/s00120-024-02515-z

Hybrid-DRGs in der Urologie

7. Beurteilung des Konzeptes der Hybrid-DRGs in der Urologie?

	sehr schlecht	schlecht	eher schlecht	indifferent	eher gut	gut	sehr gut
Wie beurteilen Sie das Konzept der Hybrid-DRGs in der Urologie?	○	○	○	○	○	○	○

8. Wie sehr stimmen Sie den folgenden Aussagen zu?

	stimme überhaupt nicht zu	stimme nicht zu	stimme eher nicht zu	indifferent	stimme eher zu	stimme zu	stimme voll und ganz zu
Die Einführung der Hybrid-DRGs erleichtert meine alltägliche Arbeit.	○	○	○	○	○	○	○
Ich finde es **gerecht**, dass derselbe Eingriff sowohl ambulant als auch stationär über die Hybrid-DRG abgerechnet werden kann.	○	○	○	○	○	○	○

9. Motivieren Hybrid-DRGs in der Urologie Sie, Ihre frühere operative Tätigkeit wieder aufzunehmen oder neu operativ tätig zu werden?

- ○ Ja
- ○ Nein
- ○ Weiß nicht

10. Denken Sie, dass die Einführung von Hybrid-DRGs in der Urologie zu einer besseren Patientenversorgung führen wird?

Ja, weil: ____________

Nein, weil: ____________

Weiß nicht ____________

11. Denken Sie, dass die Einführung von Hybrid-DRGs in der Urologie für Sie zu mehr Arbeit führen wird? Beispielsweise durch eine Verlagerung von postoperativen Maßnahmen in den ambulanten Bereich?

- ◯ Ja
- ◯ Nein
- ◯ Weiß nicht

Zusatzmaterial: https://doi.org/10.1007/s00120-024-02515-z

Hybrid-DRGs in der Urologie

12. Beurteilung des Konzeptes der Hybrid-DRGs in der Urologie?

	sehr schlecht	schlecht	eher schlecht	indifferent	eher gut	gut	sehr gut
Wie beurteilen Sie das Konzept der Hybrid-DRGs in der Urologie?	○	○	○	○	○	○	○

13. Wie sehr stimmen Sie der folgenden Aussage zu?

	stimme überhaupt nicht zu	stimme nicht zu	stimme eher nicht zu	indifferent	stimme eher zu	stimme zu	stimme voll und ganz zu
Die Einführung der Hybrid-DRGs erleichtert meine alltägliche Arbeit.	○	○	○	○	○	○	○
Ich finde es **gerecht**, dass derselbe Eingriff sowohl ambulant als auch stationär über die Hybrid-DRG abgerechnet werden kann.	○	○	○	○	○	○	○

14. Denken Sie, dass die Einführung von Hybrid-DRGs in der Urologie zu einer besseren Patientenversorgung führen wird?

Ja, weil: ________

Nein, weil: ________

Weiß nicht ________

15. Denken Sie, dass Hybrid-DRGs in Zukunft auch bei anderen Eingriffen angewendet werden können?

○ Nein

○ Weiß nicht

○ Ja (bei welchen?)

____

Zusatzmaterial: https://doi.org/10.1007/s00120-024-02515-z

Hybrid-DRGs in der Urologie

16. Einfluss der Einführung von Hybrid-DRGs auf die Weiterbildung und Versorgungssituation

	sehr negativ	negativ	eher negativ	kein Einfluss	eher positiv	positiv	sehr positiv
Inwiefern denken Sie, dass die Weiterbildung durch die Hybrid-DRGs beeinflusst werden?	◯	◯	◯	◯	◯	◯	◯
Inwiefern beeinflussen Hybrid-DRGs die Versorgungssituation Ihrer Patientinnen und Patienten?	◯	◯	◯	◯	◯	◯	◯

17. Bieten Ihrer Meinung nach Hybrid-DRGs Anreize für berufsgruppenübergreifende Zusammenarbeit und Investitionen, z.B. Investition in gemeinsame OP-Infrastruktur?

- ◯ Ja
- ◯ Nein
- ◯ Weiß nicht

Zusatzmaterial: https://doi.org/10.1007/s00120-024-02515-z

Hybrid-DRGs in der Urologie

18. Führen Sie Harnleiterspiegelungen durch?

- ◯ Ja
- ◯ Nein

Zusatzmaterial: https://doi.org/10.1007/s00120-024-02515-z

Hybrid-DRGs in der Urologie

19. Ureterenoskopien bei **Harnleitersteinen**

Wie viele **Ureterorenoskopien bei Harnleitersteinen** führten Sie/Ihre Einrichtung 2023 etwa durch?

20. Welchen Anteil **(in %)** haben Sie davon 2023 ambulant erbracht?

0 100

21. Welchen Anteil **(in %)** werden Sie voraussichtlich 2024 davon ambulant erbringen?

0 100

22. Welchen Anteil **(in %)** werden Sie davon langfristig ambulant erbringen können?

0 100

Zusatzmaterial: https://doi.org/10.1007/s00120-024-02515-z

Hybrid-DRGs in der Urologie

23. Ureterenoskopien bei **Nierensteinen**

Wie viele **Ureterorenoskopien bei Nierensteinen** führten Sie/Ihre Einrichtung 2023 etwa durch?

24. Welchen Anteil **(in %)** haben Sie davon 2023 ambulant erbracht?

0 100

25. Welchen Anteil **(in %)** werden Sie voraussichtlich 2024 davon ambulant erbringen?

0 100

26. Welchen Anteil **(in %)** werden Sie davon langfristig ambulant erbringen können?

0 100

Zusatzmaterial: https://doi.org/10.1007/s00120-024-02515-z

Hybrid-DRGs in der Urologie

27. Führen Sie Hydrozelenresektionen durch?

- ◯ Ja
- ◯ Nein

Zusatzmaterial: https://doi.org/10.1007/s00120-024-02515-z

Hybrid-DRGs in der Urologie

28. Hydrozelenresektionen

Wie viele Hydrozelenresektionen führten Sie/Ihre Einrichtung 2023 etwa durch?

29. Welchen Anteil **(in %)** haben Sie davon 2023 ambulant erbracht?

0 100

30. Welchen Anteil **(in %)** werden Sie voraussichtlich 2024 davon ambulant erbringen?

0 100

31. Welchen Anteil **(in %)** werden Sie davon langfristig ambulant erbringen können?

0 100

32. Welchen Anteil **(in %)** können Sie sich vorstellen, ab 2025 als Hybrid-DRG zu erbringen?

0 100
